# Molecular epidemiology and antimicrobial resistance of *Salmonella* isolates from broilers and pigs in Thailand

**DOI:** 10.14202/vetworld.2019.1311-1318

**Published:** 2019-08-23

**Authors:** Dusadee Phongaran, Seri Khang-Air, Sunpetch Angkititrakul

**Affiliations:** Research Group for Animal Health Technology, Faculty of Veterinary Medicine, Khon Kaen University, Khon Kaen, Thailand

**Keywords:** antimicrobial resistance, broilers, pigs, pulsed-field gel electrophoresis, *Salmonella* spp

## Abstract

**Aims:**

This study aimed to determine the prevalence and antimicrobial resistance pattern of *Salmonella* spp., and the genetic relatedness between isolates from broilers and pigs at slaughterhouses in Thailand.

**Materials and Methods:**

Fecal samples (604 broilers and 562 pigs) were collected from slaughterhouses from April to July 2018. *Salmonella* spp. were isolated and identified according to the ISO 6579:2002. *Salmonella*-positive isolates were identified using serotyping and challenged with nine antimicrobial agents: Amoxicillin/clavulanate (AMC, 30 µg), ampicillin (AMP, 10 µg), ceftazidime (30 µg), chloramphenicol (30 µg), ciprofloxacin (CIP, 5 µg), nalidixic acid (NAL, 30 µg), norfloxacin (10 µg), trimethoprim/sulfamethoxazole (SXT, 25 µg), and tetracycline (TET, 30 µg). Isolates of the predominant serovar *Salmonella* Typhimurium were examined for genetic relatedness using pulsed-field gel electrophoresis (PFGE).

**Results:**

*Salmonella* was detected in 18.05% of broiler isolates and 37.54% of pig isolates. The most common serovars were Kentucky, Give, and Typhimurium in broilers and Rissen, Typhimurium, and Weltevreden in pigs. Among broilers, isolates were most commonly resistant to antibiotics, NAL, AMP, TET, AMC, and CIP. Pig isolates most commonly exhibited antimicrobial resistance against AMP, TET, and SXT. Based on PFGE results among 52 *S*. Typhimurium isolates from broilers and pigs, a high genetic relatedness between broiler and pig isolates (85% similarity) in Cluster A and C from PFGE result was identified.

**Conclusion:**

The results revealed high cross-contamination between these two animal species across various provinces in Thailand.

## Introduction

*Salmonella* is a critical zoonotic foodborne pathogen that is a serious public health concern worldwide [1]. The pathogen is internationally recognized as the main cause of diarrheal disease that infects 10% of the population every year [2]. Non-typhoidal *Salmonella* (NTS) is a major group of *Salmonella* that causes salmonellosis in humans and animals worldwide. Most cases of human salmonellosis are caused by *Salmonella* through consumption of food contaminated by the pathogen [3].

Broilers and pigs are important reservoirs of NTS [4]. The most common NTS serovars in Thailand’s broilers are *Salmonella* Weltevreden, *Salmonella* Rissen, and *Salmonella* Corvallis [5-7]. In Thailand’s pigs, the most common serovars are *S*. Rissen and *Salmonella* Typhimurium [8-11]. Most NTS serovars are not severe pathogens because of many serovars in pigs and *Salmonella* Pullorum and *Salmonella* Gallinarum in broilers are host specific, so they cause disease in animals, but not in humans. Most humans infected with serovars leading to gastroenteritis transmitted by consuming contaminated food or environmental contact can recover without treatment. However, some serovars such as *Salmonella* Choleraesuis, *S*. Typhimurium, *Salmonella* Heidelberg, *Salmonella* Virchow, *Salmonella* Infantis, *Salmonella* Agona, and *Salmonella* Enteritidis can threaten patient lives, especially infants, elders, and immunosuppressed patients [12].

Antimicrobial-resistant NTS has become a significant problem worldwide. Antimicrobial resistance has led to the failure in the treatment of gastroenteritis patients, prolonged hospitalization, and increased medical costs, leading to massive public health, and economic impacts. Moreover, the presence of multidrug-resistant *Salmonella* (resistant to more than three antimicrobial agents) exacerbates this problem. Several studies reported that 40-80% of broilers and pigs in Thailand carry multidrug-resistant *Salmonella* isolates [9,13-16].

Pulsed-field gel electrophoresis (PFGE) is an important tool for controlling and investigating *Salmonella* outbreaks [17]. The PFGE method is used in many studies to assess the genetic relatedness of *Salmonella* between human and livestock isolates from animals of the same species in Thailand [11,15,18,19]. However, research into the genetic relatedness between *Salmonella* isolates across different animal species is still limited.

This study aimed to determine the prevalence of *Salmonella* serovars, antimicrobial resistance patterns, and genetic relationships between *Salmonella* isolates from broilers and pigs to provide more data on the dispersal of *Salmonella* among animals, the environment, and humans.

## Materials and Methods

### Ethical approval

The study was reviewed and approved by the Institutional Animal Care and Use Committee of Khon Kaen University, Thailand (IACUC-KKU-16/61).

### Sample collection

Six hundred and four cloacal swab samples from broilers and 562 rectal swab samples from pigs were randomly collected after stunning the animals at local slaughterhouses in nine provinces of Thailand (n=1,116) (Khon Kaen, Nong Khai, Chiang Rai, Chiang Mai, Lampang, Lamphun, Mae Hong Son, Phayao, and Sa Kaeo) between April and July 2018. All samples were suspended in 5 mL of Cary Blair transport media (Oxoid, England), stored in an icebox (4°C), and transported to the laboratory at the Department of Veterinary Public Health, Khon Kaen University, Khon Kaen, Thailand.

### Isolation and identification of *Salmonella*

*Salmonella* spp. isolates were purified using the standard method ISO 6579:2002. All samples were enriched in 9 mL buffered peptone water (BPW, Difco, France) for 24 h at 37°C and selected using Modified Semi-solid Rappaport-Vassiliadis medium (MSRV, Difco, France) through incubation for 24 h at 42°C. The plates with white swarming growth were subcultured on xylose-lysine deoxycholate agar (XLD, Difco, France) and incubated at 37°C for 24 h. Positive colonies (red colonies with black centers) were selected and subjected to biochemical tests to confirm *Salmonella* positive isolates using triple sugar iron agar (TSI, Difco, France) and motility indole lysine (MIL, Difco, France) for 24 h at 37°C [20]. The slide agglutination test used commercial antiserum and followed the Kauffman–White scheme [21]. It was performed for *Salmonella* spp. serovar identification (ECDC 2012; S&A Reagents Lab, Bangkok, Thailand).

### Antimicrobial susceptibility testing

The *Salmonella* isolates were tested against nine antimicrobial agents using the agar disk diffusion technique following Clinical and Laboratory Standards Institute M100 27^th^ standard: Ampicillin (AMP, 10 μg), amoxicillin/clavulanate (AMC, 30 μg), chloramphenicol (CHL, 30 μg), ciprofloxacin (CIP, 5 μg), ceftazidime (CAZ, 30 μg), nalidixic acid (NAL, 30 μg), norfloxacin (NOR, 10 μg), trimethoprim/sulfamethoxazole (SXT, 1.25/23.75 μg), and tetracycline (TET, 30 μg) [22]. *Escherichia coli* ATCC 25922 was used as the control strain.

### PFGE

*S*. Typhimurium isolates were analyzed using PFGE according to the standard operating procedure for PulseNet PFGE of *E. coli* O157:H7, *E. coli* non-O157 (STEC), *Salmonella* serotypes, *Shigella sonnei*, and *Shigella flexneri* [23]. *Salmonella* Braenderup H9812 was used as a reference marker and the isolates were digested using *Xba*I (New England Biolabs Ipswich, MA, USA). The PFGE profiles were analyzed using BioNumerics software version 7.6 (Applied Maths, Belgium) with the dice coefficient similarity index of 1% optimization and 1% tolerance using the unweighted pair group method with arithmetic means (UPGMA).

### Statistical analysis

The prevalence of *Salmonella* spp. and antimicrobial resistance patterns of broiler and pig samples were analyzed using Pearson’s Chi-square test and significance differences (p<0.05) using SPSS statistical software version 17.0 (IBM, USA).

## Results

### *Salmonella* prevalence and serovars

Among the isolates from broilers and pigs, 28.67% (320/1116) were positive for *Salmonella* spp., 37.54% (211/562) of pig rectal swab samples were positive, and 18.05% (109/604) of isolates from broiler cloacal swab samples were positive for *Salmonella* spp. The prevalence of *Salmonella* in pig samples (37.54%) was significantly higher than in broiler samples (18.05%) (p<0.001). Forty-six different serovars were identified in broiler isolates (n=31) and pig isolates (n=19), as shown in Table-1. The most common serovars in broiler isolates were *Salmonella* Kentucky (22.94%), followed by *Salmonella* Give (20.18%), *S*. Typhimurium (7.34%), *Salmonella* Mbandaka (5.50%), *Salmonella* Agona (3.67%), *Salmonella* Derby (3.67%), and *Salmonella* Singapore (3.67%). The most common serovars in isolates from pigs were *S*. Rissen (36.97%), followed by *S*. Typhimurium (21.33%), *S*. Weltevreden (14.70%), *Salmonella* Stanley (6.64%), and *S*. Agona (3.79%). The serovars *S*. Agona, *S*. Stanley, *S*. Typhimurium, and *S*. Give were found in both broiler and pig samples. The slide agglutination test showed that broiler and pig isolates both had *S*. Typhimurium as the most common of the previously detected serovars, and we used it as the main isolate for determining genetic relatedness between broiler and pig isolates using PFGE.

**Table 1:** Serotypes of *Salmonella* from pigs and broilers in Thailand.

*Salmonella* groups	*Salmonella* serotypes	Broilers n (%)	Pigs n (%)	Total n (%)
B	*Salmonella* Agona	4 (3.67)	8 (3.79)	12 (3.75)
	*Salmonella Derby*	4 (3.67)	-	4 (1.25)
	*Salmonella* Haifa	1 (0.92)	-	1 (0.31)
	*Salmonella* Heidelberg	-	1 (0.47)	1 (0.31)
	*Salmonella* Limete	-	2 (0.95)	2 (0.63)
	*Salmonella* Paratyphi B	-	1 (0.47)	1 (0.31)
	*Salmonella* Stanley	1 (0.92)	14 (6.64)	15 (4.69)
	*Salmonella* Typhimurium	8 (7.34)	45 (21.33)	53 (16.56)
C	*Salmonella* Albany	1 (0.92)	-	1 (0.31)
	*Salmonella* Altona	-	4 (1.90)	4 (1.25)
	*Salmonella* Athinai	2 (1.83)	-	2 (0.63)
	*Salmonella* Augustenborg	1 (0.92)	-	1 (0.31)
	*Salmonella* Bardo	2 (1.83)	-	2 (0.63)
	*Salmonella* Bareilly	2 (1.83)	-	2 (0.63)
	*Salmonella* Braenderup	3 (2.75)	-	3 (0.94)
	*Salmonella* Cayar	1 (0.92)	-	1 (0.31)
	*Salmonella* Chomedey	1 (0.92)	-	1 (0.31)
	*Salmonella* Corvallis	3 (2.75)	-	3 (0.94)
	*Salmonella* Cremieu	2 (1.83)	-	2 (0.63)
	*Salmonella* Dabon	1 (0.92)	-	1 (0.31)
	*Salmonella* Cyprus	1 (0.92)	-	1 (0.31)
	*Salmonella* Istanbul	1 (0.92)	-	1 (0.31)
	*Salmonella* Kentucky	25 (22.94)	-	25 (7.81)
	*Salmonella* Litchfield	1 (0.92)	-	1 (0.31)
	*Salmonella* Molade	1 (0.92)	-	1 (0.31)
	*Salmonella* Newport	1 (0.92)	-	1 (0.31)
	*Salmonella* Rissen	-	78 (36.97)	78 (36.97)
	*Salmonella* Saint pant	1 (0.92)	-	-
	*Salmonella* Singapore	4 (3.67)	-	-
	*Salmonella* Stuttgart	1 (0.92)	-	-
	*Salmonella* Mbandaka	6 (5.50)	-	-
	*Salmonella* Wippra	2 (1.83)	-	-
D	*Salmonella* Enteritidis	3 (2.75)	-	3 (0.94)
	*Salmonella* Lome	-	3 (1.42)	3 (0.94)
	*Salmonella* Panama	-	5 (2.37)	5 (1.56)
	*Salmonella* Powell	-	1 (0.47)	1 (0.31)
	*Salmonella* Victoria	-	1 (0.47)	1 (0.31)
E	*Salmonella* Assinie	-	2 (0.95)	2 (0.63)
	*Salmonella* Biafra	-	1 (0.47)	1 (0.31)
	*Salmonella* Give	22 (20.18)	1 (0.47)	23 (7.19)
	*Salmonella* Fulda	-	6 (2.84)	6 (1.88)
	*Salmonella* Ugor	1 (0.92)	-	1 (0.31)
	*Salmonella* Weltevreden	-	31 (14.70)	31 (9.69)
G	*Salmonella* Kedougou	-	5 (2.37)	5 (1.56)
I	*Salmonella* Hvittingfoss	-	2 (0.95)	2 (0.63)
O	*Salmonella* Alachua	2 (1.83)	-	2 (0.63)
	Total	109 (100)	211 (100)	320 (100)

### Antimicrobial susceptibility

Among broiler isolates positive for *Salmonella*, 64.22% were resistant to at least one antimicrobial agent and 28.44% were multidrug-resistant (MDR) (resistant to three or more antimicrobial agents). Antimicrobial resistance of broiler isolates was most commonly observed against NAL (49.54%), followed by AMP (30.28%), TET (27.52%), amoxicillin (26.61%), CIP (23.85%), NOR (19.27%), CHL (4.59%), SXT (4.59%), and CAZ (1.83%). Among pig isolates, 74.88% were resistant to at least one antimicrobial agent and 38.39% were MDR. All isolates were susceptible to NOR. Antimicrobial resistance in pig *Salmonella* isolates was most commonly observed against AMP (69.05%) and TET (66.19%), as shown in Table-2. There was no significant difference (p=0.077) in the prevalence of MDR in *Salmonella* isolates from broilers and pigs. The antimicrobial resistance rate for amoxicillin, CIP, NAL, and NOR in *Salmonella* isolates from broilers was significantly higher than that for *Salmonella* isolates from pigs (p<0.001), and the resistance of pig isolates to AMP, SXT, and TET was significantly higher than that for broiler isolates (p<0.001). In addition, there was no significant difference in the prevalence of antimicrobial resistance against CHL (p=0.155) and CAZ (p=0.447) between broiler and pig *Salmonella* isolates.

**Table 2:** *Salmonella* antimicrobial resistance percentages in broiler and pig isolates from Thailand.

Samples	Amount	Antimicrobial resistance agents (%)								

		AMC	AMP	CHL	CAZ	CIP	NAL	NOR	SXT	TET
Broilers	109	29 (26.60)	33 (30.27)	5 (4.58)	2 (1.83)	26 (23.85)	54 (49.54)	21 (19.26)	5 (4.58)	30 (27.52)
Pigs	210	2 (0.95)	145(69.04)	19 (9.04)	7 (3.33)	2 (0.95)	5 (2.38)	0	75 (35.71)	139 (66.19)

*AMP=Ampicillin, AMC=Amoxicillin/clavulanate, CHL=Chloramphenicol, CAZ=Ceftazidime, CIP=Ciprofloxacin, NAL=Nalidixic acid, NOR=Norfloxacin, SXT=Trimethoprim/sulfamethoxazole, TET=Tetracycline

Thirty-one antimicrobial resistance patterns were identified from the 320 *Salmonella* positive isolates, as shown in Table-3. The most common antimicrobial resistance patterns were NAL (21.10%) and AMC/AMP/CIP/NAL/NOR/TET (16.51%) in broiler isolates and AMP/SXT/TET (28.91%) and AMP/TET (25.12%) in pig isolates.

**Table 3:** *Salmonella* antimicrobial resistance pattern in broiler and pig isolates from Thailand.

Antimicrobial resistance pattern	Number of isolates (%)	

	Broilers (n=109)	Pigs (n=211)
AMP		14 (6.63)
CHL	4 (3.67)	
CAZ		2 (0.95)
CIP		1 (0.48)
NAL	23 (21.10)	1 (0.48)
SXT	1 (0.92)	
TET	2 (1.84)	5 (2.37)
AMP- NAL	4 (3.67)	
AMP-TET		53 (25.12)
CHL-SXT	1 (0.92)	
CIP-NAL	4 (3.67)	
SXT-TET		1 (0.48)
AMC-AMP-TET	5 (4.59)	
AMP-CHL-TET		2 (0.95)
AMP-SXT-TET		61 (28.91)
CHL-NAL-TET		1 (0.48)
CHL-SXT-TET		1 (0.48)
AMC-AMP-CAZ-CIP	1 (0.92)	
AMC-AMP-CIP-NAL	1 (0.92)	
AMC-AMP-SXT-TET	2 (1.84)	1 (0.48)
AMP-CHL-CAZ-TET		2 (0.95)
AMP-CHL-SXT-TET		9 (4.27)
AMP-CIP-NAL-TET		1 (0.48)
CAZ-CIP-NAL-NOR	1 (0.92)	
AMC-CIP-NAL-NOR-TET	1 (0.92)	
AMP-CHL-CAZ-NAL-TET		1 (0.48)
AMP-CHL-CAZ-SXT-TET		1 (0.48)
AMP-CIP-NAL-NOR-TET	1 (0.92)	
AMC-AMP-NAL-SXT-TET	1 (0.92)	
AMC-AMP-CHL-NAL-SXT-TET		1 (0.48)
AMC-AMP-CIP-NAL-NOR-TET	18 (16.51)	
Susceptible to all	39 (35.78)	53 (25.12)

AMP=Ampicillin, CHL=Chloramphenicol, CAZ=Ceftazidime, CIP=Ciprofloxacin, NAL=Nalidixic acid, SXT=Trimethoprim/sulfamethoxazole, TET=Tetracycline, AMC=Amoxicillin/clavulanate, NOR=Norfloxacin

### PFGE profiles

**Figure-1:**
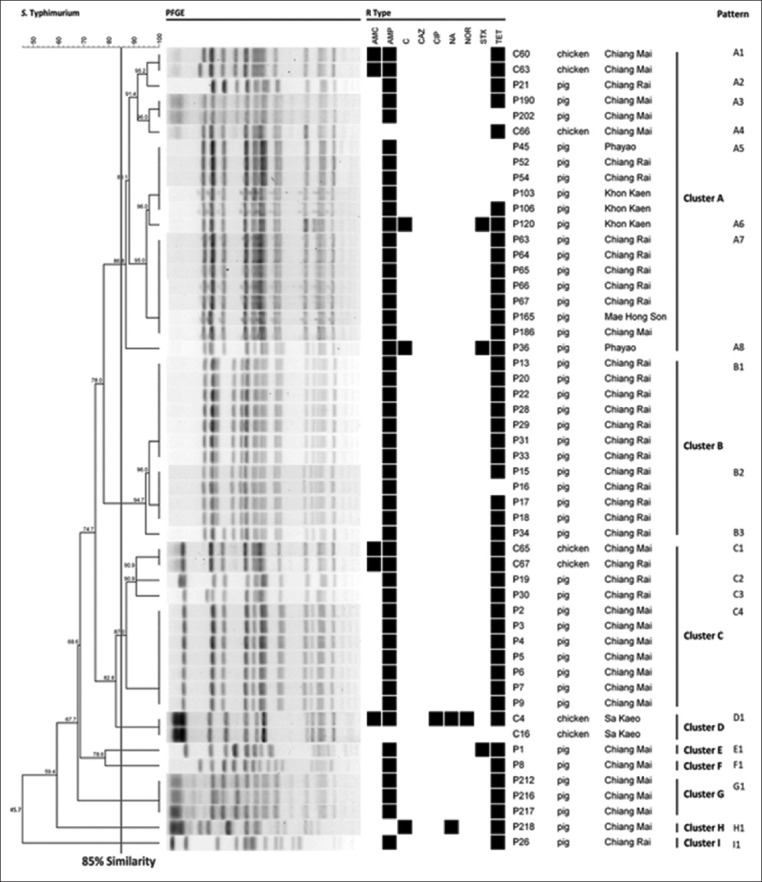
Dendrogram of 52 PFGE-*Xba*l profiles of *Salmonella* Typhimurium isolated from broilers and pigs at slaughterhouses in Thailand (n=Resistance, o=Susceptibility).

The PFGE dendrogram of 52 *S*. Typhimurium isolates from broilers (n=7) and pigs (n=45) with 85% similarity valuation is shown in Figure-1. Nine clusters (A-I) with 21 PFGE patterns were created. Cluster A was the predominant group, containing PFGE patterns with three broiler isolates collected from Chiang Mai and 17 pig isolates from Khon Kaen, Chiang Mai, Chiang Rai, Phayao, and Mae Hong Son. Cluster B consisted of three PFGE patterns from 12 pig isolates collected from Chiang Rai. Cluster C had four PFGE patterns from two broiler isolates from Chiang Mai and Chiang Rai and nine pig isolates from Chiang Mai and Chiang Rai. Cluster D contained one PFGE pattern from two broiler isolates (Sa Kaeo), and Clusters E, F, and H each had one PFGE pattern from one pig isolate (Chiang Mai). Cluster G had one PFGE pattern from three pig isolates from Chiang Mai. Cluster I had one PFGE pattern from a pig isolate from Chiang Rai.

The group of indistinguishable isolates from different provinces revealed that 80% of the isolates shared a similar antimicrobial resistance pattern (A5 pattern) composed of pig isolates from the northeastern region (Khon Kaen) and the north region (Chiang Rai and Phayao). Similar antimicrobial resistance patterns for the A7 pattern composed of pig isolates from Chiang Rai, Chiang Mai, and Mae Hong Son, and the C1 pattern of broiler isolates from Chiang Mai and Chiang Rai were also observed.

## Discussion

The prevalence of *Salmonella* spp. in pig isolates (37.54%) was significantly higher (p<0.001) than in broiler isolates (18.05%). The prevalence of *Salmonella* in broilers was lower than that reported by previous studies for Northern Thailand [24], Southern Thailand [9], Bangkok, and Central Thailand [25], but it was higher than that reported for Chiang Mai [5] and Sa Kaeo [26]. The prevalence of *Salmonella* spp. in pig isolates in this study was lower than that reported for Chiang Mai, Chiang Mai’s surrounding areas [11], and Southern Thailand [9]. However, the prevalence of *Salmonella* in pig isolates was higher than that reported for Sa Kaeo [15,26] and Central Thailand [25]. In this study, the prevalence of *Salmonella* spp. in broilers and pigs was higher than that reported for broilers and pigs from European countries: 2.35% in Belgium, 1.56% in Italy, 0% in Poland, and 11.72% in Spain from pig carcasses, and 0.26% in Belgium, 0.01% in Italy, 0.08% in Poland, and 0.06% in Spain for broiler flocks reported by food business operator [27]. As the *Salmonella* prevalence control and reduction program have not been established for broilers and pigs in Thailand, these results indicate a noticeable difference between Thailand and Europe with respect to *Salmonella* infection in food animals. This is a significant issue that Thailand should address as soon as possible. The most common serovars in broiler isolates were *S*. Kentucky, *S*. Give, and *S*. Typhimurium. This finding was inconsistent with the findings of Padungtod and Kaneene [7], who reported that the most common serovars in Thailand’s chickens were *S*. Weltevreden and *S*. Rissen. Other studies reported that *S*. Corvallis and *S*. Rissen were the most common serovars in chicken [5]. Several studies in European countries also reported that *S*. Enteritidis and *S*. Typhimurium were the most common serovars in broilers [28-30], while *S*. Rissen, *S*. Typhimurium, and *S*. Weltevreden were the most common in *Salmonella* isolates from pigs. These findings agree with several Thai studies which reported that *S*. Rissen was the most common serovar in pigs [8,9,11,19]. They are also consistent with reports from European countries that the most common serovar in pig was *S*. Typhimurium [31-33]. Another study in Thailand indicated that *S*. Weltevreden and *S*. Dumfries were the most common serovars in swine samples from Sa Kaeo [15]. These results revealed the variation of *Salmonella* spp. serovars and prevalence based on location and animal species [7].

The prevalence of MDR between broilers (28.44%) and pigs (38.39%) was not significantly different (p=0.077) in this study. However, the prevalence of MDR was high compared to other European studies, 19.70% in broiler flocks from Spain [34] and 19.30% in pigs from Denmark [33]. To deal with this issue, Thailand should adopt a more restrictive policy toward antimicrobial use in food animal production. The most common MDR pattern in the two species was different: AMC/AMP/CIP/NAL/NOR/TET for broilers and AMP/SXT/TET for pigs. Furthermore, the resistance prevalence of some antimicrobial agents differed between broiler and pig isolates. Resistance to antimicrobial agents (amoxicillin, CIP, NAL, and NOR) was higher in broiler isolates than pig isolates, but resistance to antimicrobial agents (AMP, SXT, and TET) was higher in pig isolates than broiler isolates. These differences indicated a potential difference in the use and frequency of antimicrobial agents in the broiler industry compared to the pig industry. There are few reports on antimicrobial use in food animal production in Thailand, which report the common use of amoxicillin, colistin, doxycycline, oxytetracycline, and tilmicosin in Thai poultry farms [35]. The top ten most commonly used veterinary antimicrobials in food producing animals (pigs, poultry, cattle, and aquatic animals) were found to be amoxicillin, halquinol, chlortetracycline, tiamulin, doxycycline, sulfadimidine, colistin, tilmicosin, gentamicin, and tylosin [36]. These results are consistent with the findings of our study regarding the most common antimicrobial resistance in broiler (NAL, AMP, TET, and amoxicillin) and pig isolates (AMP, TET, and SXT). The inconsistent results can be explained by the fact that the use of antimicrobial agents in Thai food animal production varies depending on the level and farm type [37]. The fluoroquinolone group is the recommended antimicrobial agent for treating severe and multidrug-resistant salmonellosis both in humans and animals [38,39]. The previous studies in Thailand reported that *Salmonella* isolates from poultry were still susceptible or resistant at a lower proportion to CIP and NOR [3,5,7,9,24,26]. However, the prevalence of broiler isolates resistant to CIP and NOR was higher here than in previous studies. The higher prevalence of resistance indicated a possible decrease in fluoroquinolone susceptibility, which may reflect an association between the high proportion of resistance to NAL (49.50%), CIP, and NOR in broiler isolates [40,41]. The NAL and fluoroquinolone-resistant *Salmonella* spp. can lead to severe *Salmonella* infections, treatment failure, or extended hospital stays for patients with multidrug-resistant salmonellosis compared to patients with susceptible *Salmonella* spp. [42].

The high genetic relatedness (85% similarity) in Clusters A and C between these two animal species and the results of PFGE of 52 *S*. Typhimurium isolates from broilers and pigs indicated high genetic relatedness between broiler and pig isolates. The identical PFGE patterns (A5 and A7) of pig isolates were collected from various provinces. Most of the isolates in each identical PFGE pattern had the same antimicrobial resistance pattern, except for P106 in A5, which revealed cross-contamination in pig isolates from Khon Kaen, Chiang Rai, and Phayao (A5), and Chiang Rai, Chiang Mai, and Mae Hong Son (A7). The cross-contamination could be explained by the following: Worker’s hands that contaminated by *Salmonella*, contaminated transport cages, personnel moving among the provinces, or flies as a transmission vector [43]. However, the high genetic relatedness between broiler and pig isolates should be further investigated to develop a deeper understanding of the epidemiological characteristics and to prevent further cross-contamination between these two animal species.

## Conclusion

This study revealed the prevalence of *Salmonella* spp. in pigs and broilers from multiple provinces of Thailand. Moreover, the problem of MDR *Salmonella* spp. and the increasing resistance to CIP and NOR compared to previous studies should be noted as a concerning public health issue. We also revealed high genetic relatedness between *Salmonella* isolates from broilers and pigs and cross-contamination in pig isolates across different provinces. These findings should be investigated further to gain a better understanding of the epidemiological characteristics and to prevent cross-contamination in food animal productions. We also obtained noticeably different prevalence and MDR resistance results in our study compared to those from European countries, which already has established control and reduction programs for *Salmonella* in poultry and pig production. We also conclude that Thailand’s government should implement new policies such as control and reduction programs for *Salmonella* to control and reduce the prevalence of *Salmonella* and MDR resistance issues in Thailand.

## Authors’ Contributions

SA conceived and designed the experiments, provided materials and reagent. SA, DP, and SK collected samples. DP performed the experiments, analyzed data and wrote the manuscript. All authors read and approved the final manuscript.
